# The Slopes Remain the Same: Reply to Wolfe (2016)

**DOI:** 10.1177/2041669516673383

**Published:** 2016-11-10

**Authors:** Árni Kristjánsson

**Affiliations:** Faculty of Psychology, University of Iceland, Reykjavik, Iceland; Institute of Cognitive Neuroscience, University College London, Reyjavik, Iceland

**Keywords:** attention, parallel processing, visual search

## Abstract

Wolfe (2016) responds to my article (Kristjánsson, 2015), arguing among other things, that the differences in slope by response method in my data reflect speed accuracy trade-offs. But when reaction times and errors are combined in one score (inverse efficiency) to sidestep speed accuracy trade-offs, slope differences still remain. The problem that slopes, which are thought to measure search speed, differ by response type therefore remains.

Recently, I argued that the distinction between preattentive and attentive processing that is often made in visual search studies, based on whether slopes of set size and response time (RT) are positive or flat, has outstayed it’s welcome and may even sometimes hamper progress (Kristjánsson, 2015).

Wolfe (2016) responded, arguing that wholesale abandonment of slopes would be unwise given their usefulness. That is a worthy cause, especially had slopes been in any danger. I did not actually argue against the use of slopes but simply highlighted the theoretical baggage they tend to carry in the visual search literature. Slopes are obviously a useful tool and can, for example, be used to measure the rate at which items are processed. Whether they do so in visual search is debatable, however, and the assumption that they actually do, and are therefore the true measures of search speed, may yield questionable conclusions.

Wolfe (2016) echoes my warnings about thinking of slopes as measures of actual cognitive mechanisms and processing levels or types. Slopes are not simple metrics of whether a search is “parallel” or “serial.” This assumption is nevertheless often made in the literature. So Wolfe and I agree that slopes are interpretable and useful but disagree on whether they have outstayed their welcome in the visual search literature.

Wolfe claims that the most challenging data for the use of slopes as measures of search rate are changes in slope when only the task is changed (present/absent vs. go/no-go). If slopes are a measure of search speed, they should not be affected by response type, which was nevertheless the case in Kristjánsson (2015). Wolfe argues that error rates increase with set size in the critical conditions that I report, and that this data involve a “classic speed accuracy trade-off [SAT].” Wolfe is right that there is evidence of SATs in the data but the important question is whether SATs account for *all* the differences in slope by response method reported in Kristjánsson (2015).

There is no single agreed upon way of assessing whether SATs account for condition differences, and a definitive way may not exist (Bruyer & Brysbaert, 2011). But any such assessment must almost certainly involve some convolution of RTs and error rates. Inverse efficiency scores (IES; Townsend & Ashby, 1978) have been used to combine RTs and error rates in one score to compensate for differences in error rates (e.g., Bruyer & Brysbaert, 2011; Vandierendonck, 2016). IES involve multiplying mean RT by error rates yielding a single score (IES = Mean RT/1 − Mean error rate). Slopes of IES and set size can then be measured. If there are still slope differences between response conditions in Kristjánsson (2015), then the problem for the RT by set size methodology remains.

Table 1 shows the results of applying IES scores to RTs and error rates in Kristjánsson (2015) and also to data from Wang, Kristjánsson, and Nakayama (2005) where a similar slope difference by response method was reported. The IES transform does not affect the patterns in the results in any fundamental way. For easy conjunction search, there are still condition differences of 5 ms per added item to the set size. This means that the search is 5 ms slower per added item for the more traditional present/absent task than the Go No-Go task. This is also the case for easy conjunction search from Wang et al. (2005). The slope differences for the difficult conjunction search are, however, smaller than in the original data. In sum, SATs do not easily account for slope differences by response method suggesting that slopes are not straightforward measures of search rate.

**Table 1.** Slope and Intercepts for Inverse Efficiency scores (in ms) from Kristjánsson (2015) and Wang et al. (2005).

	Easy conjunction search		Hard conjunction search		Feature search		Wang et al. (2005)	
Response	Intercept	Slope	Intercept	Slope	Intercept	Slope	Intercept	Slope
PA present	844	1	1051	41	679	−1	1092	4
PA absent	862	12	1362	44	756	−3	1241	8
GNG present	815	−4	906	36	589	−1	844	−1
GNG absent	809	3	1151	43	661	−1	878	1

*Note.* PA = present/absent task; GNG = Go No-Go task.

There are also notable intercept differences. Intercept differences are often ignored in visual search studies, based on the assumption that they involve a separate processing stage from the actual search (Sternberg, 1969), which also relies on the questionable assumption that slopes are the true measure of search. In any case, outright dismissal of intercept differences as irrelevant to visual search is unhelpful, but further speculation is beyond the current scope.

In the end, I do not think that Wolfe and I disagree on very much. And we agree that task-based differences in slope are a challenge to the RT × Set size methodology. We may disagree on whether SATs account for the task-based slope differences, but I think that the current analysis makes clear that they cannot easily be dismissed as SATs.

There are likely other ways of assessing SATs, but it is hard to see that they would involve anything else than taking both error rates and RTs in to account as inverse efficiency scores do, although weights assigned to each could be varied.

This issue deserves more detailed analysis. Inverse efficiency scores are not uncontroversial and carry a number of assumptions (Bruyer & Brysbaert, 2011; Vandierendonck, 2016). Recent studies highlight the usefulness of analyzing RT distributions (Antoniades et al., 2013; Burnham, Cilento, & Hanley, 2015; Kristjánsson & Jóhannesson, 2014; Palmer, Horowitz, Torralba, & Wolfe, 2011; Wolfe, Palmer, & Horowitz, 2010). Testing whether RT distributions differ by response method could shed further light on the issue. Currently, my coworkers and I are collecting large data sets with varied response methods that will enable such detailed analyses.
